# Artificial Intelligence Algorithms for Analysis of Geographic Atrophy: A Review and Evaluation

**DOI:** 10.1167/tvst.9.2.57

**Published:** 2020-10-26

**Authors:** Janan Arslan, Gihan Samarasinghe, Kurt K. Benke, Arcot Sowmya, Zhichao Wu, Robyn H. Guymer, Paul N. Baird

**Affiliations:** 1 Centre for Eye Research Australia, University of Melbourne, Royal Victorian Eye and Ear Hospital, East Melbourne, Victoria, Australia; 2 Department of Surgery, Ophthalmology, University of Melbourne, Victoria, Australia; 3 School of Computer Science and Engineering, University of New South Wales, Kensington, New South Wales, Australia; 4 School of Engineering, University of Melbourne, Parkville, Victoria, Australia; 5 Centre for AgriBioscience, AgriBio, Bundoora, Victoria, Australia

**Keywords:** age-related macular degeneration, geographic atrophy, artificial intelligence

## Abstract

**Purpose:**

The purpose of this study was to summarize and evaluate artificial intelligence (AI) algorithms used in geographic atrophy (GA) diagnostic processes (e.g. isolating lesions or disease progression).

**Methods:**

The search strategy and selection of publications were both conducted in accordance with the Preferred of the Preferred Reporting Items for Systematic Reviews and Meta-Analyses (PRISMA) guidelines. PubMed and Web of Science were used to extract literary data. The algorithms were summarized by objective, performance, and scope of coverage of GA diagnosis (e.g. lesion automation and GA progression).

**Results:**

Twenty-seven studies were identified for this review. A total of 18 publications focused on lesion segmentation only, 2 were designed to detect and classify GA, 2 were designed to predict future overall GA progression, 3 focused on prediction of future spatial GA progression, and 2 focused on prediction of visual function in GA. GA-related algorithms reported sensitivities from 0.47 to 0.98, specificities from 0.73 to 0.99, accuracies from 0.42 to 0.995, and Dice coefficients from 0.66 to 0.89.

**Conclusions:**

Current GA-AI publications have a predominant focus on lesion segmentation and a minor focus on classification and progression analysis. AI could be applied to other facets of GA diagnoses, such as understanding the role of hyperfluorescent areas in GA. Using AI for GA has several advantages, including improved diagnostic accuracy and faster processing speeds.

**Translational Relevance:**

AI can be used to quantify GA lesions and therefore allows one to impute visual function and quality-of-life. However, there is a need for the development of reliable and objective models and software to predict the rate of GA progression and to quantify improvements due to interventions.

## Introduction

Age-related macular degeneration (AMD) is the most common cause of irreversible vision loss and legal blindness (visual acuity [VA] < 6/60 in the better eye), accounting for 8.7% of blindness globally for individuals aged 50 years and older.^1^ One of the two late stages of the disease is referred to as geographic atrophy (GA). GA currently affects approximately 5 million patients worldwide (with its prevalence predicted to increase to 9 to 10 million cases by the year 2040) but its etiology remains vague and no drug therapies are currently available.^2^^–^^5^ GA is characterized by death of the retinal pigment epithelium (RPE) and photoreceptor cells, as well as loss of the underlying choriocapillaris. GA appears as sharply demarcated areas (i.e. lesions) at the macula.^3^ When atrophic lesions approach the central foveal area, visual tasks, such as reading and recognizing faces, become increasingly difficult.^6^^,^^7^ The lesions continue to grow over time, leading to irreversible vision loss. The rate of irreversible vision loss is highly variable and risk factors include demographic and environmental factors, such as age, sex, smoking, and diet.^7^ Diagnostic imaging characteristics, such as hyperfluorescent areas – bright areas that are a build-up of lipofuscin (a fluorophore) and are a precursor to lesion formation – have been suggested as markers for understanding the progression of the disease. However, recent histopathologic studies strongly suggest that vertical stacking (or clumping) of fluorophore-containing cells, such as the RPE, is a major cause of hyperfluorescent boundaries.^8^^–^^11^

Our current understanding of what drives GA progression and how to predict its growth (i.e. its progression) is still limited and strategies to measure lesion size are slow and costly in terms of human resources. Artificial Intelligence (AI) has been used extensively for “big data” analytics in the past based on electronic health records and, more recently, AI approaches have been extended to screening retinal images subsequently showing promise in diagnostics.^12^ An advantage of AI-based analysis is that it can evaluate megabytes of data very rapidly and cost-effectively.^13^ AI systems can discriminate image features and colors at a much higher resolution and greater bandwidth than humans and can therefore enhance the process of information discovery.^14^ AI can also integrate clinical information with features appearing in diagnostic images to improve classification accuracy.^15^ This is evident in radiology and dermatology, which have already been the subject of research in AI-based diagnostics, with promising results.^13^

Recently, there has been a rapid increase in the number of publications describing AI applications in ophthalmology. These have tended to focus primarily on detection of disease for screening purposes and for triaging cases for referral. The ultimate aim being to allow rapid assessment of disease with minimal human intervention and increased throughput. Particular focus has been primarily on ocular diseases, such as diabetic retinopathy and glaucoma.^16^^–^^18^

In this article, we present an overview of currently available AI algorithms that have been used for the automation or evaluation of GA rather than as a screening approach. The algorithms were summarized by (a) their objective (e.g. lesion automation or classification), (b) their performance (e.g. level of accuracy), and (c) whether the algorithms covered the entire scope of GA diagnosis (e.g. if the current AI algorithm combined lesion automation with predictive modeling to understand the progression of GA). In addition to summarizing the literature, important gaps have been identified and discussed. This paper also describes advantages of AI in GA diagnosis, and discusses possible future directions in research.

## Methods

### Eligibility Criteria and Search Terms for Review

The search strategy and selection of publications were conducted in accordance with the Preferred Reporting Items for Systematic Reviews and Meta-Analyses (PRISMA) guidelines.^19^ Literary sources included PubMed and Web of Science.^20^^,^^21^ Grey/manual search techniques were additionally used (i.e. screening the list of references of found publications) to ensure complete coverage of articles for this review. No time limitations were imposed on the search. Table 1 outlines the inclusion and exclusion criteria. Two authors (J.A. and G.S.) conducted the search to ensure accuracy and reproducibility in search results. The search was concluded on August 28, 2020, to ensure the list of publications were up-to-date and complete.

**Table 1.** Inclusion and Exclusion Criteria for the Literature Review

Inclusion Criteria	Exclusion Criteria
Original, peer-reviewed publication that assessed an AI-based algorithm for GA	Systematic reviews, meta-analyses, narrative reviews
Published in English language	Reviews with unsystematic methods
No limitation on time frame	Editorials, opinion pieces, and commentary letters
No limitation on study design or study population	Publications in which GA was not the only disease/disease state under assessment (e.g. a classification algorithm that classified AMD into neovascular AMD or GA)
Could include conference proceedings and abstracts	Publications that developed an algorithm which could have a widespread use in ophthalmology (e.g. vessel or drusen segmentation not specifically designed for GA assessment)

AI, artificial intelligence; AMD, age-related macular degeneration; GA, geographic atrophy.

The following search terms were used: geographic atrophy [AND] artificial intelligence; geographic atrophy [AND] progression [AND] artificial intelligence; geographic atrophy [AND] machine learning; geographic atrophy [AND] deep learning; geographic atrophy [AND] feature extraction; geographic atrophy [AND] computer vision; hyperfluorescence [AND] artificial intelligence; hyperfluorescence [AND] artificial intelligence [AND] geographic atrophy; hyperfluorescent [AND] artificial intelligence; hyperfluorescent [AND] artificial intelligence [AND] geographic atrophy; RPE atrophy [AND] artificial intelligence; cRORA; cross-validated prediction [AND] geographic atrophy; automated [AND] geographic atrophy.

### Study Selection Process

**Figure 1.**
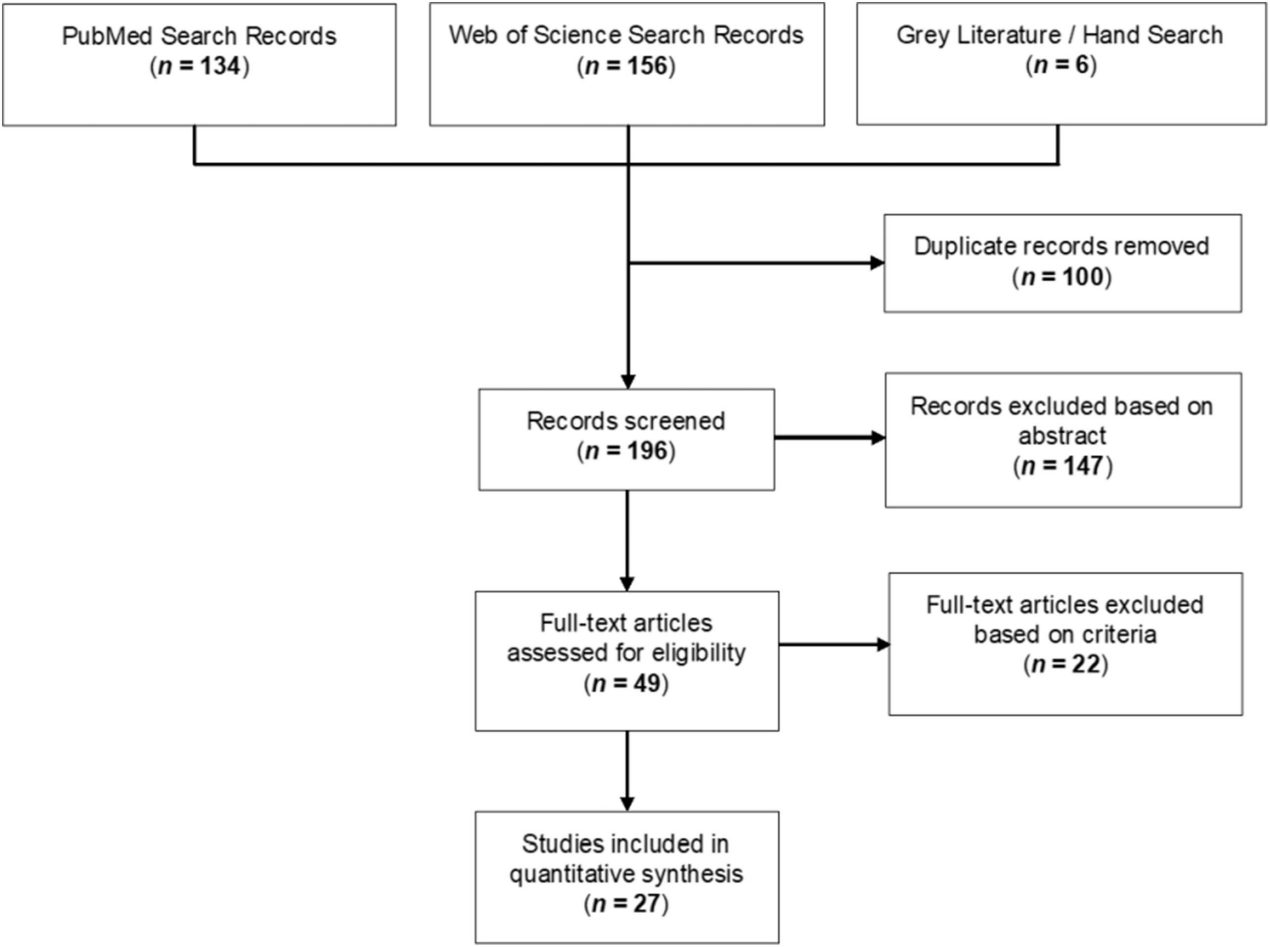
The PRISMA flowchart illustrating the literature selection process. Online databases, PubMed and Web of Science, were used for this review. Reference lists from identified publications were also reviewed to identify any GA-AI papers which may have been missed using our search keywords. AI, artificial intelligence; GA, geographic atrophy.

The objective was to identify AI applications that were specifically designed for understanding or diagnosing GA. Generalized ophthalmic AI applications (e.g. vessel segmentation, which could be used across multiple ocular diseases), and publications that treated GA as a subset rather than the primary focus (e.g. classification of various stages of AMD generally) were excluded. A total of 27 assessable publications were found (Fig. 1).

### Data Collection and Analysis

Data collection focused on the following variables: the objective of the study, retinal image modality used, sample size, summary of AI techniques used, whether the algorithm was compared to a human grader (and, if so, the number of graders used for comparison), and finally the results of the study. Table 2 summarizes the data collected and the reasons for each variable's collection.

**Table 2.** Measured Variables Collected for Review

Measured Variable	Reasoning
**Study objective**	Recording the objective of each study allowed the quantification of the intention (and current direction) of GA-AI studies. For example, what is the primary purpose of GA-AI studies currently? Is it to understand progression or simply to automate current annotative processes?
**Retinal image modality**	This variable quantified the various image types used in GA-AI studies to highlight (1) what common imaging modalities are used in GA assessment, and (2) do different image types contribute to more or less successful AI applications? Although there are several imaging modalities available, the FAF is considered an appropriate tool to measure GA size and growth rate longitudinally with a high degree of reproducibility.^22^
**Total sample size**	The general consensus in AI and statistical theory is that the larger the sample size the more accurate the algorithm. However, large sample sizes may be difficult to attain in medical research, depending on the disease prevalence, confidentiality, and ethical and privacy concerns. This variable summarizes the sample sizes used for GA-AI algorithms, and by extension, whether sample sizes of tens and hundreds would suffice in developing highly accurate algorithms.
**Artificial intelligence algorithms used**	This variable assessed the algorithms used, and whether there was a diversity of methods employed or whether similar AI algorithms were being used repetitively.
**Human grader comparison**	Human grader comparison refers to comparing a proposed AI method to that of the current gold standard in GA diagnostics: the human. The expectation is that an AI algorithm should be designed to meet or exceed grading by a human grader. This variable identified publications that have evaluated the accuracy of their algorithms against a human grader, and whether the AI was successful in meeting and/or exceeding expectations.
**Study outcomes**	This variable quantified the diagnostic accuracy of the proposed GA-AI algorithms.

AI, artificial intelligence; FAF, fundus autofluorescence; GA, geographic atrophy.

## Results

A total of 290 results were found in PubMed and Web of Science; 100 of these results were duplicates, leaving a total of 190 original publications for further assessment (see Fig. 1). Six additional publications were found using grey/hand search methods, which included searching through references of found articles and identifying publications that may have been missed with the literature search. The total publications increased to 196 papers.

The abstracts of all publications found were first reviewed and used to conduct initial screenings for suitability. These screenings resulted in the exclusion of 147 publications, in accordance with the exclusion criteria in Table 1. A further scrutiny of the remaining 49 publications found only 27 AI papers that solely focused on GA, while the 22 remaining publications used AI to assess GA in combination with other ocular conditions (e.g. classify the different stages of AMD, from early to late). The final 27 publications were included in the quantitative assessment. A synopsis of the literature can be found in Table 3.^23^^–^^49^ The complete review dataset, which included in-depth information regarding all computing processes used and results obtained, can be found in Supplementary Table S1.

**Table 3.** Synopsis of GA-AI Publication Techniques

Category	Reference	Retinal Image Modality	Total Sample Size	Artificial Intelligence Algorithm	Human Grader Comparison	Outcome Measures (Examples)
1. Detection and classification of GA	Treder et al., 2018^23^	FAF	690 images	Deep CNN using TensorFlow (Google Inc.)	No	Sensitivity, specificity, accuracy
	Keenan et al., 2019^24^	CFP	59,812 images (from AREDS)	CNN	Yes	
2. Segmentation of GA	Deckert et al., 2005^25^	FAF	40 eyes	Region-growing algorithm	No	DSC
	Lee et al., 2008^26^	FAF	100 images	Watershed transform algorithm	No	
	Devisetti et al., 2011^27^	FAF and IR	N/A	Supervised neural network with scaled conjugate gradient learning algorithm	No	
	Chen et al., 2013^28^	SD-OCT and FAF	**Dataset 1:** 55 scans**Dataset 2:** 56 scans with corresponding FAF	Geometric active contour model	Yes	
	Hu et al., 2013^29^	SD-OCT and FAF	20 eyes	Level set method for segmentation	Yes	
	Hu et al., 2014^a^^,^^b^^30^	FAF	16 images from 16 patients	Supervised pixel classification using *k*-nearest neighbor	Yes	
	Ramsey et al., 2014^31^	CFP and FAF	Ten patients, each with an average of three image pairs	Fuzzy *c*-Means segmentation	Yes	
	Feeny et al., 2015^32^	CFP	143 images	Random forest decision tree	No	
	Hu et al., 2015^33^	FAF	16 eyes	Supervised pixel classification using *k*-nearest neighbor	Yes	
	Niu et al., 2016^34^	SD-OCT and FAF	**Dataset 1:** 55 scans**Dataset 2:** 56 scans with corresponding FAF	Chan-Vese model via local similarity factor	Yes	
	Fang et al., 2017^35^	SD-OCT	117 volume scans from 39 participants	CNN-graph search model	Yes	
	Hu et al., 2018^a^^,^^b^^36^	FAF	50 images	CNN	Yes	
	Hu et al., 2018^a^^,^^b^^37^	IR	70 images from 70 subjects	CNN	Yes	
	Ji et al., 2018^38^	SD-OCT	**Dataset 1:** 51 scans**Dataset 2:** 54 scans	Sparse autoencoders deep network	Yes	
	Xu et al., 2018^b^^39^	SD-OCT	**Dataset 1:** 55 SD-OCT cube scans from 8 patients**Dataset 2:** 56 SD-OCT cube scans from 56 patients	3D CNN	Yes	
	Yang et al., 2018^a^^,^^b^^40^	SD-OCT and FAF	N/A	Region-growing algorithm	Yes	
	Wu et al., 2019^41^	SD-OCT and synthesized FAF	56 SD-OCT volumes from 56 patients	Region-aware adversarial network to synthesize FAF images and U-Net for segmentation	No	
	Xu et al., 2019^42^	SD-OCT	**Dataset 1:** 55 scans**Dataset 2:** 56 scans with corresponding FAF images	A two-stage learning model with offline- and self-learning based on stacked sparse auto-encoders	Yes	
3. A. Prediction of future overall GA progression	Pfau et al, 2019^43^	FAF and IR	296 eyes of 201 patients	Linear mixed-effects model	No	MAE, MSECoefficient of determination (*R^2^*)
	Liefers et al., 2020^44^	CFP	**Development and evaluation:** Total of 409 images from Blue Mountains Eye Study and the Rotterdam Study (87 from BMES and 322 from RS)**Application to assess GA growth:** 3589 images from AREDS	Eight-level encoder-decoder network and linear regression	Yes	
3. B. Prediction of future spatial GA progression	Niu et al., 2016^45^	SD-OCT	118 SD-OCT scans from 38 eyes in 29 patients	Chan-Vese model for segmentation and random forest for prediction	No	DSC
	Pfau et al., 2020^46^	FAF, IR, SD-OCT, and OCTA	98 eyes and 59 patients	Mixed-effect logistic regression	No	
	Schmidt-Erfurth et al., 2020^47^	SD-OCT and FAF	491 SD-OCT volumes from 87 eyes of 54 patients	Residual U-Net and linear regression	No	
4. Prediction of visual function in GA	Künzel et al, 2020^48^	FAF, IR, and SD-OCT	87 patients	Linear regression modelling and LASSO for multicollinearity	No	MAE, MSE, *R^2^*
	Pfau et al., 2020^49^	FAF, SD-OCT and IR	41 eyes from 41 patients (from the Directional-Spread-in-GA (DSGA) study)	Random forest decision tree	No	

a Abstract only information.

b Conference paper.

N/A, not applicable or information is missing.

AI, artificial intelligence; CFP, color fundus photograph; CNN, convolutional neural network; DSC, Dice Similarity Coefficient; FAF, fundus autofluorescence; GA, geographic atrophy; IR, near-infrared imaging; MAE, Mean Absolute Error; MSE, Mean Squared Error; SD-OCT, spectral domain optical coherence tomography.

### Summary of Literature

**Figure 2.**
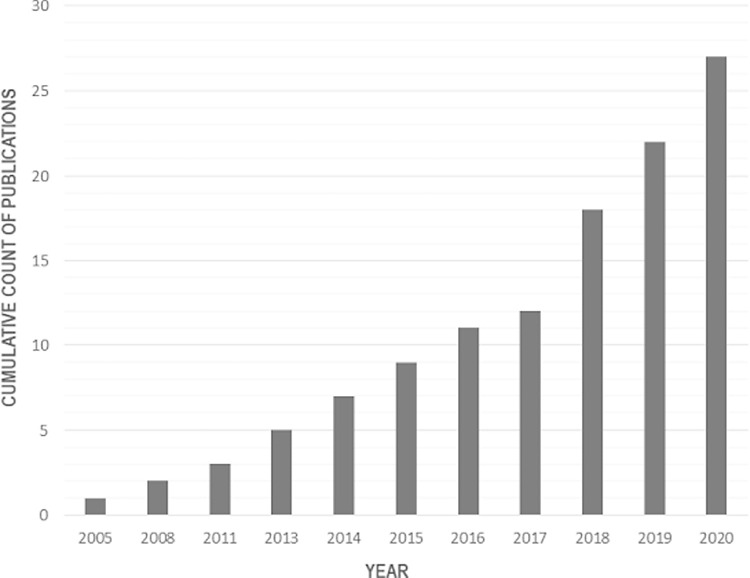
Cumulative count of GA-AI publications. There is an increasing trend of GA-AI publications. AI, artificial intelligence; GA, geographic atrophy.

A 2005 paper by Deckert et al. was the earliest publication found in the GA-AI space (Fig. 2). Of the 27 publications found, 18 were dedicated to GA segmentation only (e.g. lesions or retinal layers that explain GA), 2 focused on the detection and classification of GA, 2 assessed overall GA progression (with one including segmentation as well), 3 assessed spatial GA progression (with 2 including segmentation), and finally 2 assessed visual function prediction in GA. No publications were found that discussed other aspects of automating GA, such as the automated extraction of hyperfluorescent areas, although some, such as Pfau et al.,^43^ did assess hyperfluorescent phenotypes in the modeling process. Sample sizes ranged from 16 to 59,812 images with the latter being a subset of images from the Age-Related Eye Disease Study (AREDS) dataset and used by Keenan et al. (2019)^24^ for the detection and classification of GA-related models. This image dataset used by Keenan and colleagues was the largest dataset used with all other publications using tens or hundreds of images.

**Figure 3.**
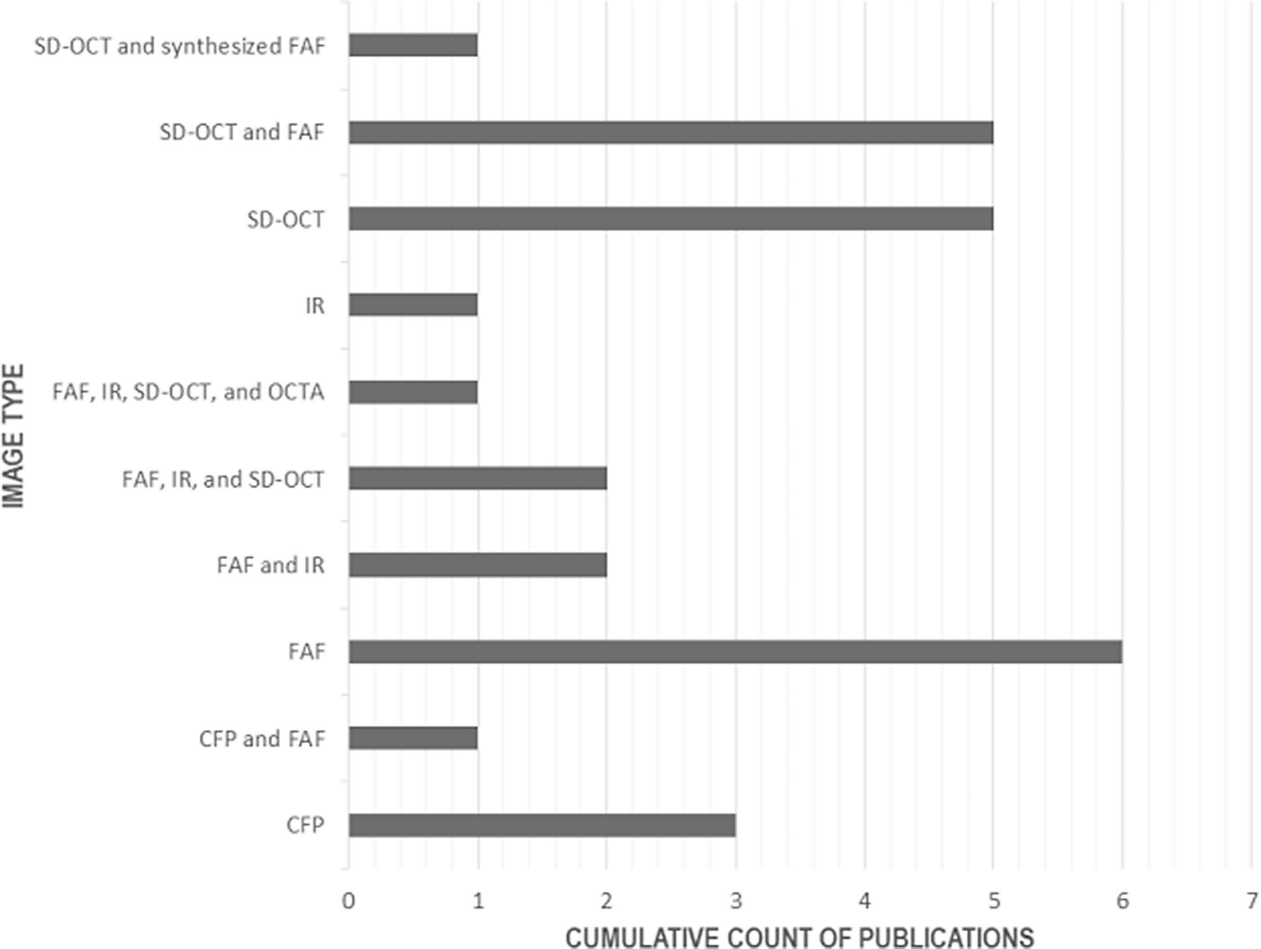
Imaging modalities used in GA-AI studies. FAF only images were the most commonly used image modality among GA-AI studies (*n* = 6). A combination of SD-OCT and FAF imaging (*n* = 5) and SD-OCT only (*n* = 5) were the second most commonly used imaging types in GA-AI studies, followed by CFP only (*n* = 3). AI, artificial intelligence; GA, geographic atrophy; CFP, color fundus photograph; FAF, fundus autofluorescence; IR, near-infrared; SD-OCT, spectral domain optical coherence tomography; OCTA, optical coherence tomography angiography.

A range of diagnostic tools were available to evaluate the severity of GA. The diagnostic tools used and reported in the 27 manuscripts include: stereoscopic color fundus photography (CFP), fundus autofluorescence (FAF), near-infrared (IR) FAF and the spectral domain optical coherence tomography (SD-OCT), to name a few.^7^^,^^50^ FAF imaging was the most commonly used in GA-AI publications (*n* = 6; Fig. 3). This was followed by a combination of SD-OCT and FAF (*n* = 5), SD-OCT only (*n* = 5), and CFP only (*n* = 3).

Fifteen of the 27 publications evaluated the performance of their algorithms against the current gold standard – human expert graders. It was noted that several tools existed to augment and improve current human processes and using a human grader as a benchmark to evaluate the success of an AI system was a sensible approach. The remaining 12 publications did not compare their algorithms to a human grader.

### Category 1: Comparison of GA Detection and Classification Algorithms

Only 2 of the 27 identified publications were focused on the detection and classification of GA. Treder et al. developed three classification models using FAF images and deep convolutional neural networks (CNNs): GA versus healthy, GA versus other retinal diseases, and finally a GA classifier relating to diffuse-trickling (i.e. a rapidly progressing autofluorescence phenotype, which was previously shown to exhibit a distinct genetic risk profile).^23^^,^^51^ Their training accuracies ranged from 0.98 to 0.99, whereas validation accuracies ranged from 0.77 to 0.96. Keenan et al.^24^ generated three binary GA-related classification models using CFP and CNN: a GA detection, a central-GA detection, and a centrality detection model. These models had an accuracy of 0.965, 0.966, and 0.762, respectively. The human grader had an accuracy of 0.975. Keenan and coworkers thus demonstrated an instance where an AI algorithm fell short of the human grader.

### Category 2: Comparison of GA Segmentation Algorithms

**Figure 4.**
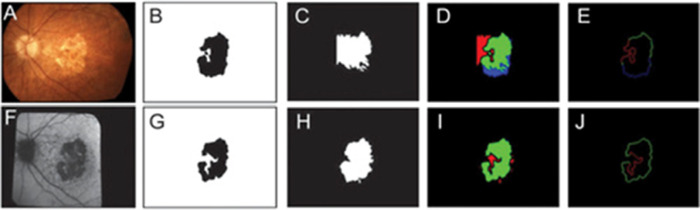
Examples of image segmentation using the Fuzzy *c*-Means algorithm reported by Ramsey *et al*.^31^ Top row illustrates CFP-based segmentation and the bottom row FAF-based segmentation. B and G are ground truths, C and H are segmentation results, D and I are color coded maps of segmentation results, and E and J illustrate which GA borders were correctly identified (i.e. *green*). See also the website of MathWorks (https://www.mathworks.com/discovery/image-segmentation.html) for many other examples of image segmentation.

Eighteen GA segmentation-only algorithms were found in the literature. These segmentation algorithms focused on isolating the GA lesions from various retinal images. There are several benefits to such a process, including improving upon current human annotation methods, which can be both tedious and time-consuming. These algorithms could also be a stepping-stone in automating the documentation of GA progression. The following algorithms were used in segmenting GA (see Table 3): region-growing (segmentation based on similarity of color intensities), interactive segmentation using watershed transform (changes image features for easier detection of regions of interest), level set approach (shape and contour-based segmentation), geometric active contour model (extraction of objects from an image), Fuzzy *c*-means (a clustering method), *k*-nearest neighbor (*k*NN; finds objects nearest to query by calculating a distance metric), the Chan-Vese model via local similarity factor (identifies objects with no clear boundaries), CNN (identifies features of interest by convolution filtering and a neural network), sparse autoencoder deep networks (an unsupervised learning model) and an offline/self-learning model (elements of learning are known to the learner), eight-level encoder-decoder network (the encoder is pretrained for classification and decoder simply using the encoder to discriminate features), and a modified residual U-Net (a popular biomedical segmentation architecture that incrementally varies the number of learning filters). For a more detailed description of the processes and results of each publication, refer to Supplementary Table S1. Figure 4 shows examples of the segmentation operation using the Fuzzy *c*-means algorithm.^31^

For published segmentation algorithms, the sensitivity ranged from 0.47 to 0.983, the specificity ranged from 0.929 to 0.99, and the accuracy ranged from 0.42 to 0.995 (Table 4). The lower sensitivity of 0.47 and accuracy of 0.42 were the results of the Ramsey et al.^31^ paper when their Fuzzy *c*-Means algorithm was applied to CFPs. The correlation coefficient ranged from 0.82 to 0.9979. Generally, these algorithms demonstrated good agreement when compared to human graders or other available commercial software. For example, the sparse encoder deep network by Ji et al.^38^ outperformed the segmentation accuracies of two human graders (e.g. a higher correlation coefficient of 0.986 [algorithm] versus 0.970 [grader 1] and 0.979 [grader 2] for dataset 1). Furthermore, the Dice Similarity Coefficient (DSC) - a spatial overlap index and a reproducibility validation metric that measures the agreement between results obtained using the gold standard human grader and the machine-predicted results^32^^,^^52^ – ranged from 0.68 to 0.89 for segmentation-only algorithms. The DSC is the appropriate metric for assessment of segmentation performance because it quantifies the degree of match between ground truth and machine output.

**Table 4.** Segmentation-Only Algorithm Outcomes

Summary of Findings for Segmentation-Only Algorithms (*n* = 18)^a^	
**Sensitivity range**	0.47–0.983
**Specificity range**	0.93–0.99
**Accuracy range**	0.42–0.995
**Mean overlap ratio range**	0.659–0.899
**Correlation coefficient range**	0.82–0.998
**Dice similarity coefficient range**	0.68–0.89
**Positive predictive value range**	0.79–0.87
**False discovery rate range**	0.13–0.20

a These results represent a total of 18 publications.

### Category 3A: Prediction of Future Overall GA Progression

Two publications discussed *overall* GA progression: Pfau et al. and Liefers et al.^43^^,^^44^ Pfau and colleagues evaluated shape-descriptive factors on lesion progression and they quantified this using a linear mixed-effects model with a two-level random effect (i.e. eye- and patient-specific effects). Assessed variables included lesion area, perimeter, and circularity and were normalized using the square root transformation. The coefficient of determination, *R^2^*, was used as the outcome measure. Models were assessed for two scenarios: (1) predicting progression of an unknown patient, and (2) prediction of future progression with previous observations from a patient. For their combined model (i.e. model with all relevant variables included), they achieved an *R^2^* of 0.244 for scenario 1 and 0.391 for scenario 2. Liefers et al. described a segmentation component (i.e. eight-level encoder-decoder network) and a growth model using linear regression. Liefers and colleagues also assessed association of shape features with GA growth rate. Their segmentation model achieved a maximum DSC of 0.72 ± 0.26 (*n* = 315). They found nine structural biomarkers - area, filled area, convex area, convex solidity, eccentricity, roundness, foveal involvement, perimeter, and circularity – which were significantly associated with growth rate (*P* ≤ 0.05).

### Category 3B: Prediction of Future Spatial GA Progression

Three publications used AI to predict future spatial GA progression: Niu et al., Pfau et al., and Schmidt-Erfurth et al.^45^^–^^47^ Niu et al. and Schmidt-Erfurth et al. both combined segmentation with progression modeling. Niu et al.^45^ utilized their previously published Chan-Vese model and added a random forest with 100 trees to build its prediction model using 19 extracted features. They created three potential prediction models: (1) a prediction of growth at first follow-up visit using baseline features trained from the general patient data, (2) prediction of growth for every visit using baseline and first follow-up visit features trained from the general patient data, and (3) prediction of growth from the third visit onward using baseline and first follow-up visit using the same patient's data. The DSCs presented for three models were divided into two further sections: prediction with current GA regions (i.e. DSCs of 0.81 ± 0.12, 0.84 ± 0.10, and 0.87 ± 0.06) and prediction excluding current GA regions (i.e. DSCs of 0.72 ± 0.18, 0.74 ± 0.17, and 0.72 ± 0.22). Sensitivities across the 3 models were 0.81 ± 0.16, 0.86 ± 0.13, and 0.90 ± 0.09, respectively, whereas specificities were 0.97 ± 0.02, 0.96 ± 0.04, and 0.95 ± 0.05. Correlation coefficients of enlargement rate were 0.87, 0.74, and 0.72, respectively. Schmidt-Erfurth and colleagues used a residual U-Net for their segmentation and a linear regression for their progression modeling.^47^ Results from the segmentation were not available. They found that hyper-reflective foci (HRF) concentration was positively correlated with GA progression in unifocal and multifocal GA (all *P* < 0.001) and de-novo GA development (*P* = 0.037). Local progression speed correlated positively with local increase of HRF (*P* value range < 0.001–0.004). Global progression speed, however, did not correlate with HRF concentrations (*P* > 0.05). Changes in HRF over time did not have an impact on the growth in GA (*P* > 0.05). Pfau et al. categorized eyes into three diagnostic groups: (1) retinal pigment epithelium atrophy with treatment-naïve quiescent choroidal neovascularization (CNV); (2) retinal pigment epithelium atrophy with a history of exudative type 1 CNV; and (3) retinal pigment epithelium atrophy without evidence of CNV. Using their pixel-wise extracted features, both localized and global progressions were assessed. A mixed-effects logistic regression model was fitted for localized progression, which was then followed up with a global progression using point-wise (mixed-effects) model. They found that localized presence of treatment-naïve quiescent type 1 CNV was associated with markedly reduced odds for the localized future progression of RPE atrophy (odds ratio [OR] = 0.21; 95% confidence interval [CI] = 0.19–0.24; *P* < 0.001). Localized presence of exudative type 1 CNV was associated with markedly reduced odds for the localized future progression of RPE atrophy (OR = 0.46; 95% CI = 0.41–0.51; *P* < 0.001). Their model performed at a DSC of 0.87 (95% CI = 0.85–0.89) when all topographic locations were considered.^46^

### Category 4: Prediction of Visual Function in GA

Two publications focused on the visual functions associated with GA. Künzel et al. studied the association of vision-related quality of life (VRQOL) and visual function/structural biomarkers in GA.^48^ Their final model was obtained by fitting a linear model to the complete dataset at baseline using LASSO regression (to account for multicollinearity). With the outcome set to VRQOL, they found predictors such as best-corrected visual acuity of the better eye, low-luminance visual acuity (LLVA) for the better eye, GA size, foveal sparing status, and LLVA for the worst eye yielded a model with an *R*^2^ of 0.32. Pfau et al.^49^ identified predictors of retinal sensitivity based on the retinal microstructure in the presence of GA for their predictive model that used random forest models with 1000 trees. For their outcome metrics, they used the mean absolute error (MAE), which served as a prediction accuracy measure. They found that retinal sensitivity was predicted with an MAE of 4.64 dB for mesopic, 4.89 dB for DA cyan, and 4.40 dB for DA red testing in the absence of patient-specific data. Partial addition of patient-specific sensitivity data to the training sets decreased the MAE to 2.89 dB, 2.86 dB, and 2.77 dB. For all three types of testing, the outer nuclear layer thickness constituted the most important predictive feature (35.0%, 42.22%, and 53.74% including mean squared error [MSE]).

### Comparison of Results Between Specific Imaging Modalities

Outcomes were separated into image modality categories of FAF, SD-OCT, and CFP, irrespective of the type of algorithm used (Table 5). Several publications used multiple imaging modalities, but typically separated their results for each image type assessed. Only one publication (Devisetti et al.) did not discern its outcomes for FAF and IR (i.e. overall sensitivity 0.825 and specificity 0.93 presented).^27^ Most diverse metrics for assessment were found in FAF and SD-OCT publications, whereas fewer metrics were found to be used in the assessment of CFP. Accuracies were one of the more commonly used metrics across all imaging modalities. Accuracies for FAF ranged from 0.75 to 0.97, 0.986 to 0.955 for SD-OCT (although these were for two datasets from the same Ji et al. study), and 0.42 to 0.966 for CFP. The more suitable metric, the DSC, appeared in seven publications that used FAF, SD-OCT, and CFP. For the FAF, the DSC range was 0.83 to 0.89, 0.81 to 0.87 for SD-OCT, and 0.66 to 0.72 for CFP, suggesting that producing more agreeable results with CFP is challenging.

## Discussion

The application of AI to GA assessment has the potential to (a) improve the delivery of health care in ophthalmology by enhancing diagnostic support, (b) identify factors responsible for the development of GA by analysis of large datasets, (c) identify underlying patterns of GA growth and variability, and (d) support the development of metrics to assess interventions needed to arrest GA progression.

The review reported here revealed that the primary focus in the literature on AI in GA was on the segmentation of GA lesions (i.e. 18 of 27 publications). Two publications were found that discussed the detection and classification of GA, two that assessed overall GA progression, three that evaluated spatial GA progression, and two that predicted visual function in GA.

### Segmentation Performance

Rather than classifying an entire image, segmentation involves the isolation of different regions of interest within the image itself for the purpose of further analysis or classification. The type of segmentation often studied in GA-AI publications is called *semantic* segmentation, where regions of interest are isolated and given a label or assigned to a category.

For the evaluation of semantic segmentation, a common and appropriate metric is a similarity metric, referred to as the DSC. The DSC is a spatial index that measures the agreement between human and machine results (i.e. the degree of match in the overlap between the machine-generated output and the output based on human annotations).^53^ An alternative metric is the Jaccard index, which is positively correlated with the DSC. However, quantitatively, the Jaccard index penalizes instances of bad classification more severely than the more intuitive DSC.^54^ This may be an issue when scoring average performance across *k*-fold cross-validations.

Other common statistical metrics for assessment include sensitivity and specificity. In the context of segmentation, using specificity will help to identify the presence of over-segmentation (i.e. detecting insignificant boundaries within an image, which could lead to the segmentation of non-lesion areas). Sensitivity, on the other hand, will help to identify issues with under-segmentation (i.e. the clumping of individual segments into one when it should instead be separate).

In this review, we found the DSC was utilized in 7 of the 27 publications and applied. Hu et al. used the DSC as a metric for both the FAF and SD-OCT images in their study (i.e. FAF DSC = 0.89 ± 0.07, SD-OCT DSC = 0.87 ± 0.09) using a level set method for segmentation.^29^ Another paper by Hu and colleagues again used the DSC (twice for two test outcomes) for a supervised pixel classification algorithm using *k*NN.^30^ Both tests were based on FAF images, and produced DSCs of 0.84 ± 0.06 and 0.83 ± 0.07, respectively. Feeny et al. obtained a DSC of 0.68 ± 0.25 for their random forest algorithm using CFPs.^32^ Liefers et al. achieved a maximum of 0.72 ± 0.26 for their eight-level encoder-decoder network using their CFP development and evaluation dataset.^44^ Niu et al. used DSC to measure predicted GA regions in their three tested scenarios for SD-OCT images; their results were 0.81 ± 0.12, 0.84 ± 0.10, and 0.87 ± 0.06, respectively.^45^ Wu et al.'s segmentation algorithm resulted in a DSC of 0.872 ± 0.066 for SD-OCT and synthesized FAF images using their region-aware adversarial network to synthesize FAF images and U-Net for segmentation.^41^ Finally, Pfau et al. used the DSC metrics to assess predicted and observed atrophy and achieved a model DSC of 0.87 (95% CI = 0.85–0.89) for regions not previously affected by atrophy.^46^

When assessing algorithms based on the DSC metric alone, the performance of CFP-driven algorithms falls short of the performance achieved by human graders or algorithms that utilize more GA-friendly imaging modalities, such as the FAF and SD-OCT. For example, Liefers et al. showed that human graders outperformed their algorithm with an average human DSC of 0.78 ± 0.24 in their development and evaluation dataset. Table 5 further validates this by illustrating better metric outcomes for FAF and SD-OCT images as compared to CFP images. For example, when evaluating the studies based on the more commonly known and used metric of accuracy, we note that the range of accuracies of FAF-based algorithms were 0.75 to 0.97, 0.986 to 0.995 for SD-OCT (although both of these results were from the same study by Ji et al.^38^), and 0.42 to 0.966 for CFP.

**Table 5.** Algorithm Outcomes for Main Image Types: FAF, SD-OCT, and CFP

Evaluation Metric	FAF^a^ (n = 18)	SD-OCT^b^ (n = 14)	CFP^c^ (n = 4)
**Sensitivity range**	0.87–0.983	0.81–0.90	0.47–0.782
**Specificity range**	0.93–0.98	0.95–0.97	0.729–0.99
**Accuracy range**	0.75–0.97	0.986–0.995	0.42–0.966
**Mean overlap ratio range**	0.659–0.79	0.726–0.899	–
**Correlation coefficient range**	0.937–0.99	0.72–0.998	–
**Dice similarity coefficient range**	0.83–0.89	0.81–0.87	0.66–0.72
**Positive predictive value range**	0.80–0.87	0.83–0.86	0.82
**Negative predictive value range**	–	0.96–0.97	0.95
**False discovery rate range**	0.13–0.20	–	–
**Mean absolute error range**	2.77–4.89	2.77–4.89	–

a These results represent a total of 18 publications that have assessed FAF, including FAF only, SD-OCT and FAF, CFP and FAF, FAF and IR, and FAF, IR, and SD-OCT. In studies with combination modalities, most studies separated results based on image set. Other publications did not discern results between FAF and the other imaging modalities (e.g., Devisetti et al.^27^ used FAF and IR and stated a sensitivity of 0.825 and specificity 0.93).

b These results represent a total of 14 publications that have assessed SD-OCT, including SD-OCT only, SD-OCT and FAF, and SD-OCT, FAF and IR. Both accuracies were from Ji et al.^38^ (one for each dataset used in the study). Sensitivity, specificity, positive predictive value, and negative predictive value ranges were all from Niu et al.^45^ One study by Schmidt-Erfurth et al. is not presented, as the results were presented as various correlation *P* values.^47^

c These results represent a total of 4 publications that have assessed CFP, including CFP only and CFP and FAF.

A comparison of how image type can affect algorithm performance is described in a study by Ramsey et al. (which cited a low accuracy of 0.42; Table 5).^31^ Ramsey and colleagues used the Fuzzy *c*-Means segmentation method for CFP and FAF images; the accuracy of 0.42 ± 0.25 was associated with using this algorithm on CFP images, whereas FAF images outperformed on the same algorithm with an accuracy of 0.75 ± 0.16. The variability in performance between images trained on the same algorithms was predominantly related to the appearance of GA features in different imaging modalities. GA lesions and hyperfluorescent areas are much more evident in grayscale image types. CFP have been widely used for measuring GA lesions and are the historical gold standard for imaging GA as well as being the primary modality of measuring for large epidemiologic studies and disease classification systems. In CFP, GA lesions are seen as depigmentation of the retina, which then makes the underlying choroid more visible. However, CFP are limited in its illustration of certain GA features due to media opacities and low contrast between atrophic areas and the intact retina, thus making the detection of GA lesions and their boundaries difficult. Highly qualified and experienced clinicians and graders could find it challenging to identify GA features in CFP, thus there is some degree of intersubject variability.^55^ Due to image quality, the CFP modality is not well suited for use by automated or semi-automated detection algorithms used to distinguish between a lesion and background in the retina, with mixed results reported in the literature.^7^^,^^38^^,^^56^ Imaging modalities, such as the FAF, provide a better picture of GA status, given that these images can capture lesions and hyperfluorescent areas more clearly, and provide a better visual depiction of the state of the retina in GA-affected patients. The high contrast between atrophic and non-atrophic regions in FAF images results in more precise delineation and segmentation of GA lesions, relative to CFP images, with superior identification and reproducibility for both humans and AI algorithms.^55^

Moussa et al. compared MultiColor, CFP, FAF, IR, and SD-OCT in evaluating GA.^57^ They found that MultiColor and FAF showed the greatest intergrader agreement for GA area measurements, whereas SD-OCT showed the highest intergrader agreement of foveal involvement. The authors tabulated difficulties encountered when analyzing GA limits and foveal sparing across different images. They found that contrast-related issues were most prevalent in CFP and IR imaging, whereas MultiColor was the imaging modality most prone to artifacts. The authors concluded that the high intergrader agreement achieved by FAF relative to other imaging modalities is explained, in part, to the superior contrast. However, FAF images are dependent on xanthophyll pigment, which can be misinterpreted as atrophic areas.

The only CFP-based publication with a high metric outcome was found in the classification and detection publication by Keenan et al.^24^ Using a DeepSeeNet – a deep learning framework for grading CFPs using AREDS simplified severity scale – for three binary classification models (i.e. GA model, central GA model, and centrality detector model), Keenan and colleagues were able to achieve accuracies of 0.965 (95% CI = 0.959–0.971), 0.966 (95% CI = 0.957–0.975), and 0.762 (95% CI = 0.725–0.799), respectively. These improved achievements could be attributable to (a) the algorithm being a classifier rather than a segmentation process, which would require more easily definable borders of lesions, and (b) the very large dataset of 59,812 CFPs from 4582 participants used in the study. The image dataset used by Keenan and colleagues is the largest dataset reported, exceeding other studies by a factor of 100.

The success of Keenan and colleagues is encouraging. Due to small sample sizes, availability of medical images and data access are normally limited.^58^ Despite this, the other classification and detection paper by Treder et al.^23^ – which used Deep CNN and Tensorflow – also provided encouraging measures, including higher accuracy in its use but with only 690 FAF images. For their GA versus healthy classifier, Treder et al. achieved a training accuracy of 99% and a validation accuracy of 96%. This trend continued for their GA versus other retinal disease classification (training accuracy of 98%, and validation accuracy of 91%) and diffuse-trickling GA classifier (training accuracy was 99%, and the validation accuracy was 77%). AI processing speed would have added to the information content in these papers. For example, the task of grading a single retinal image may take a human grader 90 minutes, whereas an AI application could complete the same task in approximately 1.4 minutes.^59^ The speed of AI coupled with such accuracies provides added benefit in real-world clinical settings.

Among the 27 publications identified, 5 evaluated progression (2 for overall and 3 for spatial progression). These same publications additionally utilized segmentation and/or AI-based feature extractions as part of their progression analyses. Liefers et al. used an encoder-decoder network for segmentation of lesions from CFPs in combination with progression analysis using linear regression.^44^ The linear regression model was developed from features extracted from segmented GA areas at baseline. The extracted features included area, perimeter, number of lesions, and circularity. The dataset included 409 images from the Blue Mountains Eye Study (BMES) and the Rotterdam Study (RS; these images were classed as “development and evaluation” images) and 3589 images from AREDS, which were used to test the application of the developed algorithm in the assessment of GA progression. Their segmentation technique achieved a moderate DSC of 0.72 ± 0.26 (*n* = 315) on the BMES/RS data, and 0.66 ± 0.27 (*n* = 50) on the AREDS data.

The paper by Liefers et al. described image segmentation followed by progression analysis, where the trend was characterized by fitting a quadratic growth model up to a GA area of 12 mm^2^, but then showed significant divergence. The *R*^2^ values of 11 individual features indicated that the most significant feature associated with progression was the area of the lesion (*P* < 0.001). Additionally, the model was built using a forward selection process, which added features that yielded the highest increase in adjusted *R^2^* value. Künzel et al. also used a stepwise forward selection process for a linear regression model, using the Akaike Information Criterion (AIC) – a metric for model comparison using a measure of similarity of the expected predictive performance.^60^ There are several issues to consider with stepwise variable selection, including the selection of “important” variables and the potential problem of over-fitting to noisy data.^61^ It was conjectured by Liefers et al. that a new study of GA progression would add further knowledge - if the FAF imaging modality was used, which may be a more appropriate method of tracking GA progression because of improved image quality.

Schmidt-Erfurth et al. utilized a segmentation process, and then characterized GA progression using linear regression.^47^ A custom-built algorithm based on the residual U-Net was used for the semantic segmentation of HRF voxels (volumetric pixels) to investigate the growth of GA, using SD-OCT and FAF images. The authors used the Spearman correlation coefficient to investigate the associations between HRF concentrations and GA growth. They reported statistically significant *R* values and concluded that increased HRF concentration in the junctional zone together with progressive macular atrophy “may represent progressive migration and loss of retinal pigment epithelium.”

GA spatial progression was also investigated by Niu et al. and Pfau et al.^45^^,^^46^ Niu et al.^45^ coupled their previously formulated Chan-Vese model along with a random forest with 100 trees to build 3 potential prediction models using 19 extracted features from a dataset of 118 SD-OCT scans from 38 eyes of 29 patients. Performance metrics used were DSC, sensitivity, specificity, positive predictive values, and negative predictive values (PPVs and NPVs, respectively). The authors conducted paired *U* test analysis to compare GA predicted and observed outcomes. The DSCs presented by Niu and colleagues is encouraging, and the highest DSC presented (0.87 ± 0.06) almost matches the SD-OCT DSC of Hu et al. (0.87 ± 0.09).^29^ The DSC presented by Niu and colleagues also exceeds those presented by Liefers et al., demonstrating once again that imaging modality is just as crucial as the AI technique utilized. The paired *U* test showed a lack of statistical significance across all three testing scenarios, illustrating no statistically significant difference between the predicted and observed outcomes.

Pfau and colleagues categorized eyes into three diagnostic groups and assessed both localized and global progressions with AI-extracted features, such as pixel-wise locations.^46^ They fitted a mixed-effects logistic regression for localized progression, followed by a global progression using point-wise (mixed-effects) model, and found that both localized presence of treatment-naïve quiescent type 1 CNV and localized presence of exudative type 1 CNV were both associated with markedly reduced odds for the localized future progression of RPE atrophy (OR = 0.21 and 0.46, respectively; *P* < 0.001). The DSC was 0.87 (95% CI = 0.85–0.89) when all topographic locations were considered.

The papers by Liefers and Schmidt-Erfurth illustrate the use of statistical techniques with reliance on *P* values as a benchmark for testing for significance. The use and significance of *P* values in research has been the subject of an ongoing debate. Some suggestions have been made to change the *P* value threshold from 0.05 to 0.005 for statistical significance to ensure better repeatability and reproducibility among studies and to lessen the priority of the *P* value in research.^62^ In contrast to the latter studies, the papers by Niu and Pfau, evaluated associations of potential predictor variables, as well as evaluating the performance of their respective prediction models. Niu et al., for example, ranked the importance of 19 features and evaluated the predictive power of their model using DSC, sensitivity, specificity, and correlation coefficients. It would also be feasible to replace the *U* test used, which is also *P* value reliant, with other measures, such as the Mean Absolute Percentage Error (MAPE).^63^

### Gaps in the Literature

The publications described in this survey covered lesion segmentation, detection and classification, and progression. The models presented for GA segmentation, and the features extracted, were considered significant based on their *P* values. But the *R^2^* values indicated that associations between GA progression and image features were not always very strong and could be further investigated and validated by new or improved models. We were unable to identify publications that used AI to conclusively explain our understanding of GA in the context of time-series progression and the factors which contribute most strongly to its progression. Additionally, while linear or quadratic models were suggested in some progression studies, the appropriateness of these models was not exhaustively tested by statistical techniques. Model structure uncertainty can be tested by using a standard dataset and comparing a range of models based on goodness-of-fit metrics.^64^

The focus of publications was predominantly on lesions representing GA. No publications were identified when specifically searching for hyperfluorescence-based studies in the GA-AI spectrum. There still appears to be a knowledge gap mainly in relation to spatial GA progression. Lesion progression has been investigated using a linear mixed-effects model and the FAF phenotype (i.e. various hyperfluorescent patterns) as a feature in a cross-validated model, revealing low predictive value compared to shape-descriptive factors.^43^ The potential role of hyperfluorescence in the manifestation and progression of GA has been assessed previously.^43^^,^^65^ Simple predictive models were used, rather than AI algorithms, and thus the evaluation of hyperfluorescence association with GA progression is a neglected area of GA-AI research.

The publications described in this review have a strong emphasis on lesion segmentation, and a minor emphasis on characterization of the trend for GA progression. A future role of AI could include the identification of a universal and complete prediction model for the rate of GA progression, which would be available for multiple imaging modalities and would support the assessment of objective metrics for targeted interventions.

A final unmet need of GA-AI research is the presence of multicollinearity. Collinearity refers to closely correlated variables (e.g. HRF and FAF phenotype). Multicollinearity refers to correlations between more than two variables. Its presence can lead to biased estimations and variance inflation. Collinearity can exacerbate problems with variable selection, particularly when stepwise selection methods are used. In stepwise selection methods, the exclusion of closely related variables is arbitrary. Therefore, vital variables may be accidentally removed while insignificant variables kept.^61^^,^^66^ Multicollinearity may be overcome using AI. For example, Dumancas et al. compared 12 machine learning algorithms for handling multicollinearity amongst lipid clinical data. These techniques included partial least squares-discriminant analysis (PLS-DA), artificial neural network, LASSO, gradient boosting, random forest, and support vector machine.^66^ Results from their study found the PLS-DA to be the most suitable. The same machine learning algorithms could be similarly tested for GA progression data. For example, Künzel et al. used LASSO in order to address multicollinearity.^48^ LASSO, along with several other potential algorithms, could be similarly tested in the context of GA progression. The most suitable algorithm identified through such studies could, as suggested by Dumancas et al., be used as an automated and pre-processing technique in GA prediction modeling.

### Future Directions and Conclusion

The application of AI to the analysis of GA has a number of advantages that will support and enhance the performance of human experts. AI is capable of producing performance as a diagnostician that is comparable with that of human graders, based on classification accuracy, sensitivity, and specificity. Additionally, automated algorithms are very fast, orders of magnitude faster than humans, and can therefore provide support to clinicians and graders facing rapidly increasing demands on medical services, especially in the developing world. Furthermore, algorithms are very cost-effective, with software that can be distributed online and incorporated into the instrumentation, with continuous updating possible for ongoing improvements in performance. This has implications for telemedicine, where the algorithm could be either remotely accessed or stored on a mobile phone or cloud-based as an application. AI algorithms can provide results that are more reproducible and reliable than human graders because they are data-driven and objective, rather than subjective in nature, and therefore help to compensate for human measurement errors.

Current AI applications are largely defined by machine learning and deep learning for detection and classification using a database of images from patients. There is potential for using AI, in combination with statistical and mathematical modeling, to develop prediction models for the rate of GA progression, and to expedite discovery of objective metrics for assessment of medical interventions. This may involve the design of new experiments combining clinical data, pathology tests, and imagery.

The mechanisms of GA progression in an image may be different locally in the case of a single lesion versus globally (multiple sites in the retina) and therefore different models may be appropriate. There is a need to consider more sophisticated uncertainty analysis with respect to sources of experimental error that may be epistemic in nature rather than due to only statistical variability in measurements. Finally, in addition to grey-level intensity maps, image analysis over a range of different wavelengths is likely to add further to information discovery.

## Supplementary Material

Supplement 1
